# Cervical carcinomas that overexpress human trophoblast cell-surface marker (Trop-2) are highly sensitive to the antibody-drug conjugate sacituzumab govitecan

**DOI:** 10.1038/s41598-020-58009-3

**Published:** 2020-01-22

**Authors:** Burak Zeybek, Aranzazu Manzano, Anna Bianchi, Elena Bonazzoli, Stefania Bellone, Natalia Buza, Pei Hui, Salvatore Lopez, Emanuele Perrone, Paola Manara, Luca Zammataro, Gary Altwerger, Chanhee Han, Joan Tymon-Rosario, Gulden Menderes, Elena Ratner, Dan-Arin Silasi, Gloria S. Huang, Masoud Azodi, Peter E. Schwartz, Alessandro Santin

**Affiliations:** 1 Department of Obstetrics, Gynecology, and Reproductive Sciences, Yale School of Medicine, New Haven, CT, 06510 USA; 2 Department of Pathology, Yale University School of Medicine, 06510 New Haven, CT, USA; 3 Department of Experimental and Clinical Medicine, Magna Graecia University, Catanzaro, 88100 Italy

**Keywords:** Oncology, Oncogenesis

## Abstract

Human trophoblast cell-surface marker (Trop-2) is a surface glycoprotein originally identified in human placental tissue and subsequently found to be highly expressed by various types of human epithelial solid tumors. We investigated the efficacy of sacituzumab govitecan, an antibody-drug conjugate (ADC) comprised of a humanized anti- Trop-2 antibody, conjugated with active metabolite of irinotecan (SN-38), on Trop-2 positive cervical cancer cell lines and a xenograft model. Trop-2 expression was evaluated in 147 primary cervical tumors by immunohistochemistry, real-time polymerase chain reaction, and flow cytometry. For *in vitro* experiments, two Trop-2 positive (CVX-8, ADX-3), and one Trop-2 negative (ADX-2) cell lines were used. A cell line with a strong Trop-2 expression (CVX-8) was used to test *in vivo* antitumor activity in xenografts models. Out of 147 primary cervical cancers, 113 were squamous cell carcinomas (SCCs), and 34 were adenocarcinoma/adenosquamous carcinomas. Moderate to strong diffuse staining was seen in 95% (108/113) of SCCs, and 81% (29/34) of adenocarcinoma/adenosquamous cancers on immunohistochemistry. Trop-2 positive cell lines were highly sensitive to sacituzumab govitecan *in vitro*, with IC_50_ values in the range of 0.18 to 0.26 nM (p = 0.02, and p = 0.04 for CVX-8, and ADX-3, respectively). In xenografts, a significant tumor growth inhibition was seen after twice-weekly intravenous administration of the drug for three weeks (p < 0.0001, and p = 0.001 for sacituzumab govitecan vs naked antibody, and sacituzumab govitecan vs control-ADC, respectively). Overall survival at 90 days was significantly improved in the sacituzumab govitecan group (p = 0.014). In conclusion, sacituzumab govitecan may represent a novel targeted therapy option in cervical cancer patients overexpressing Trop-2.

## Introduction

Recent advances in treatment of cancer have not had a major impact in cervical cancer. It is the fourth most frequent cancer in women across the world after breast, colorectal, and lung with estimated 569,847 new cases and 311,365 deaths annually^1^. In 2019, it is estimated that there will be 13,170 new cases in the US and an estimated 4,250 people will die of this disease^2^. The frontline therapy after initial diagnosis is either surgery or a combination of chemotherapy and radiation depending on the stage and the patient factors^3^. Although the overall 5-year survival rate is reported to be 65.8%, 8% to 26% of the patients will develop recurrence within the first two years after completion of primary treatment, and prognosis especially for recurrent/metastatic disease still remains poor^2,4^.

Decades of experience with conventional chemotherapy in cancer treatment has proven that there is compelling need for newer biologic agents due to significant unwanted side effects and narrow therapeutic window of these drugs. Antibody-drug conjugates (ADCs) represent a new class that combines a surface receptor-targeting antibody linked to a cytotoxic molecule^5^. Ideally, the antigens that are targeted by ADCs are expressed at minimal levels on healthy tissues, but are highly expressed on tumor cells, which will lead to selective delivery and internalization of the drug to tumor cells^6^. Furthermore, the chemical structure of the linkers (non-cleavable vs cleavable) provides unique characteristics to the different ADCs^5,6^. ADCs with non-cleavable thioether linkers should be internalized and degraded in lysosomes to manifest their anti-tumor activity^6^. Because of the strong stability of the linker, these ADCs show no effect on surrounding antigen negative cells (bystander effect), but they demonstrate greatest efficacy in tumors that have high and homogenous expression of the target antigen instead^6^. On the other side, ADCs with cleavable linkers can kill not only the antigen-positive target cells but also the surrounding antigen-negative cells; these ADCs are more suitable for treatment of tumors with heterogeneous antigen expression^6^.

Sacituzumab govitecan (IMMU-132, Immunomedics) is an ADC that consists of a humanized anti-trophoblast cell surface antigen (Trop-2) antibody, conjugated with active metabolite of irinotecan (SN-38) through the cleavable CL2A linker that is subject to time dependent hydrolysis supporting the bystander effect in the tumor environment^7^. Trop-2 is a transmembrane calcium signal transducer that is highly expressed by diverse epithelial solid tumors and has been shown to be a prognostic marker in many of these cancers^8^. Low expression levels in normal healthy tissues make Trop-2 a suitable target for ADCs in cancer treatment^8^. Sacituzumab govitecan has shown promising activity in a recent phase I/II trial including patients with triple negative breast cancer^9^, hormone receptor-positive (HR+)/human epidermal growth factor receptor 2-negative (HER2-) breast cancer^10^, urothelial cancer^11^, small cell^12^ and non-small cell lung cancers^13^. Several registrational clinical trials are also underway in patients with metastatic urothelial cancer (TROPHY U-01; NCT03547973), triple-negative breast cancer (ASCENT; NCT02574455), and HR + /HER2- breast cancer (TROPICS-02; NCT03901339)

In this study, we evaluated the expression levels of Trop-2 in cervical cancer tissues and in primary cervical cancer cell lines and also examined the preclinical anti-tumor activity of sacituzumab govitecan in Trop-2 positive primary cervical cancer models and xenografts.

## Materials and Methods

### Establishment of primary cervical cancer cell lines

Approval for this study was obtained through the Yale School of Medicine Institutional Review Board and all methods were carried out in accordance with relevant guidelines and regulations. Primary cervical cancer cell lines from 9 patients were established after all patients signed consents per institutional guidelines prior to tissue collection. Informed consent was obtained from all subjects or, if subjects are under 18, from a parent and/or legal guardian. Tumors were staged according to the International Federation of Gynecology and Obstetrics (FIGO) 2009 staging system. Patient characteristics of these cell lines are summarized in Supplementary Table S1. The cell lines were established from fresh tumor biopsy samples as described previously^14–16^. Briefly, solid tumors were processed by mechanical disruption in an enzymatic solution of 0.14% collagenase type I (Sigma-Aldrich, St. Louis, MO, USA) and 0.01% DNAse (Sigma-Aldrich, St. Louis, MO, USA) in RPMI 1640 (Gibco, Life Technologies, Grand Island, NY, USA). The resulting solution was then incubated for 45 minutes at room temperature while being stirred. Mechanically and enzymatically disrupted tissue samples were then washed once with RPMI 1640 10% FBS, once with PBS, and then were plated in Petri dishes using keratinocyte-SFM (1×) (Thermo Fisher Scientific, Waltham, MA, USA), supplemented with 5 ng/mL epidermal growth factor and 35 to 50 μg/mL bovine pituitary extract (Invitrogen, Grand Island, NY), 1% penicillin with streptomycin (Mediatech, Manassas, VA), and 1% amphotericin (Life Technologies, Carlsbad, CA). The cell lines were kept in an incubator at 37 °C with 5% CO_2_ and continually monitored for growth. Primary cell lines with limited passages (<50) were used for the experiments.

### Tissue microarray

A retrospective, stage I-IV cervical cancer cohort represented in a tissue microarray (TMA) format, was used in this study (N = 147). In brief, representative areas from primary tumors were selected in hematoxylin/eosin–stained preparations by a pathologist. Formalin-fixed paraffin-embedded TMAs included 0.6 mm tissue cores from cervical carcinomas (duplicate cores for each case), which were retrieved over a 14-year period at our institution. Tissue sections were cut at 5 μm and purified goat polyclonal antibody against the recombinant human Trop-2 extracellular domain (R&D Systems, Inc., Minneapolis, MN; diluted 1:100) was applied for 1 hour. A secondary biotinylated anti-goat antibody (Vector Laboratories, Burlingame, CA; diluted 1:250) and the streptavidin–biotin complex (StreptABComplex/HRP, Dako, CA) were applied, then 303-diaminobenzidine (Dako) was used as chromogen and the sections were counterstained by hematoxylin (Dako)^17^. Appropriate negative and positive controls were used. The percentage of tumor cells with membranous Trop-2 immunoreactivity was estimated and the staining intensity was assessed semi-quantitatively as follows: 0, no staining; 1 + , weak; 2 + , moderate and 3 + , strong staining. The final immunoreactivity score was calculated by multiplying the staining intensity (1+, 2+, 3+) with the percentage of positive tumor cells, and was classified in 4 ordinal categories: 0–9 negative (score 0), 10–99 weak (score 1), 100–199 moderate (score 2), and 200–300 strong (score 3). Specific consents or waivers under an approved Yale Human Investigation committee protocol were obtained prior to processing any tissues.

### Determination of Trop-2 expression in primary cervical cancer cell lines

Trop-2 expression was analyzed by using flow cytometry in primary cervical cancer cell lines after being cultured *in vitro* for less than 50 passages. In brief, cell lines were incubated with 2.5 μg/mL of naked antibody hRS7 IgG for 2 hours at 4 degrees Celsius. A fluorescein isothiocyanate-conjugated goat anti-human F(ab1)2 immunoglobulin (FITC) was used as a secondary reagent for staining (BioSource International, Camarillo, CA). Analysis was conducted with a FACScalibur, using Cell Quest software (BD Biosciences, San Diego, CA). Data analysis for mean fluorescence index (MFI) was performed using Cell Quest (BD Biosciences) and Prism 7.01. The authenticity of any human cell lines used in this study has been proven by whole exome sequencing.

### Drugs

Sacituzumab govitecan (hRS7-CL2A-SN-38), non-targeting control ADC (h679-CL2A-SN-38), and naked antibody hRS7 IgG were obtained from Immunomedics, Inc. For the *in vitro* experiments, 2 μM stock solutions of sacituzumab govitecan and control ADC were prepared after dissolving these agents in sterile 0.9% sodium chloride as a stock solution. The drug-to-antibody ratio (DAR) of sacituzumab govitecan was 6.78, and that of control ADC was 6.84. The dosage of the drug was adjusted according to the DAR for the *in vitro* experiments, in order to deliver equivalent quantities of SN-38 to the cells treated with sacituzumab govitecan and control ADC. For the *in vivo* experiments, both sacituzumab govitecan and control ADC were dissolved in sterile 0.9% sodium chloride as a 5 mg/mL solution without a dose adjustment, as small differences would not affect the results. hRS7 IgG (molecular weight: 150 kDa) was obtained as a 10 mg/mL solution.

### Flow-cytometry based viability assays

Cervical cancer cell lines were plated in six-well tissue culture plates at a density of 60,000–110,000 cells/well in keratinocyte-SFM (1×) media supplemented with Epidermal Growth Factor 1–53 (EGF 1–53), bovine pituitary extract (BPE), 1% penicillin/streptomycin, and 1% amphotericin. After a 24-hour incubation period at 37 °C, 5% CO_2_, cells were treated with sacituzumab govitecan and hRS7 at concentrations of 0.05, 0.5, 1, 2, 4, and 10 nM. The control ADC’s concentration was adjusted according to DAR to deliver equivalent quantities of SN-38. Then the cells in the six-well plates were incubated for 72 hours. After this time period, well contents were collected in their entirety, centrifuged and stained with propidium iodide (2 µL of 500 µg/mL stock solution in PBS). The viable cells were then quantified using flow-cytometry as mean ± SEM relative to untreated cells as 100% viable controls. A minimum of 3 independent experiments per cell line was performed to determine IC_50_ of sacituzumab govitecan, control ADC, and hRS7.

### Test for antibody-dependent cell-mediated cytotoxicity (ADCC)

Standard 4-hour chromium (^51^Cr) release assays were performed to measure the cytotoxic reactivity of Ficoll–Paque-PLUS (GE Healthcare, Kings Park, NY, USA) separated peripheral blood lymphocytes (PBLs) from several healthy donors against primary cervical cancer cell lines at effector to target ratio (E:T) of 5:1 and 10:1. The release of ^51^Cr from target cells was measured as evidence of tumor cell lysis after exposure of the tumor cells to a concentration of 2.5 µg/mL of sacituzumab govitecan, control ADC, or hRS7 IgG. Tumor cells incubated with PBLs without an ADC were used as negative controls. Chimeric anti-CD20 mAb rituximab (2.5 μg/mL) was used in all bioassays as a negative control for hRS7. As a positive control condition, 1% sodium dodecyl sulfate (SDS) was used to achieve complete lysis of target cells. A gamma radiation counter (2470 WIZARD2 Automatic Gamma Counter, PerkinElmer) was used to count the ^51^Cr released from the target cells. ADCC of sacituzumab govitecan, control ADC, or hRS7 IgG was calculated by the following formula: % cytotoxicity = 100 × (*E − S)/(T − S)*, where *E* is the experimental release, *S* is the spontaneous release by target cells, and *T* is the maximum release by target cells lysed with 0.1% SDS. Results are mean ± standard error of mean (SEM).

### Bystander effect

Briefly, a 1:1 ratio of 2 + Trop-2 expressing cervical cancer cells (i.e., CVX8) and Trop-2 negative uterine serous cancer cells (i.e., ARK4) stably transfected with a Green Fluorescence Protein (GFP) plasmid (pCDH-CMV-MCSEF1-copGFP, a gift from Dr. Simona Colla, MDACC), were mixed (40,000 cells/well of each cell type) and plated in six-well plates (2 mL/well). After an overnight incubation, cells were treated with sacituzumab govitecan or isotype control ADC. After 72 hours, cells were collected, centrifuged and stained with propidium iodide (2 µL of 500 µg/mL stock solution in PBS) to identify percentages of live/dead cells in each well. Flow cytometry based assay was performed to quantify live cells as a mean ± SEM relative to untreated cells. Bystander effect was then assessed by comparing the percentage of live Trop-2 negative (ARK4) cells when they are co-cultured with Trop-2 positive cells (CVX8) and treated with control ADC versus sacituzumab govitecan.

### *In vivo* testing

A Trop2 + representative cell line (CVX8) was injected into 5–6 week old severe combined immunodeficiency (SCID) mice subcutaneously (ENVIGO, Indianapolis, IN). A total of 8 million cervical cancer cells suspended in 300 µL of a 1:1 solution of sterile PBS and Matrigel® (BD Biosciences) were injected to each mouse. Once the tumor volume reached 0.2 cm^3^, the mice were randomized into four groups (4 mice/group): saline control, sacituzumab govitecan, control ADC and hRS7. Sacituzumab govitecan, control ADC, and hRS7 were given at the dose of 500 μg IV twice per week for three weeks i.e., day^1,4,8,11,15,18^. Tumor volume was measured twice weekly and obtained with the following formula: (A^2^ x B)/2. A was the smaller perpendicular tumor diameter and B represented the largest tumor diameter size. At the end of the study, the animals were humanely euthanized. Animal care and euthanasia were carried out according to the rules and regulations as set forth by the Institutional Animal Care and Use Committee (IACUC) of Yale University. Study protocols were presented and approved by IACUC.

### Statistical analysis

The differences in the inhibition of proliferation after exposure to treatments and tumor volume differences at specific time points were evaluated by student t-test. Overall survival data was analyzed using the Kaplan-Meier method. Survival curves were compared using the log-rank test. A two-sided p-value < 0.05 was considered to be significant. Graph Pad Prism version 7 (GraphPad Software, Inc. San Diego, CA) was used for all the analyses. The data that support the findings of this study are available from the corresponding author upon reasonable request.

## Results

### Trop-2 expression in cervical cancer patient samples by TMA

**Figure 1:**
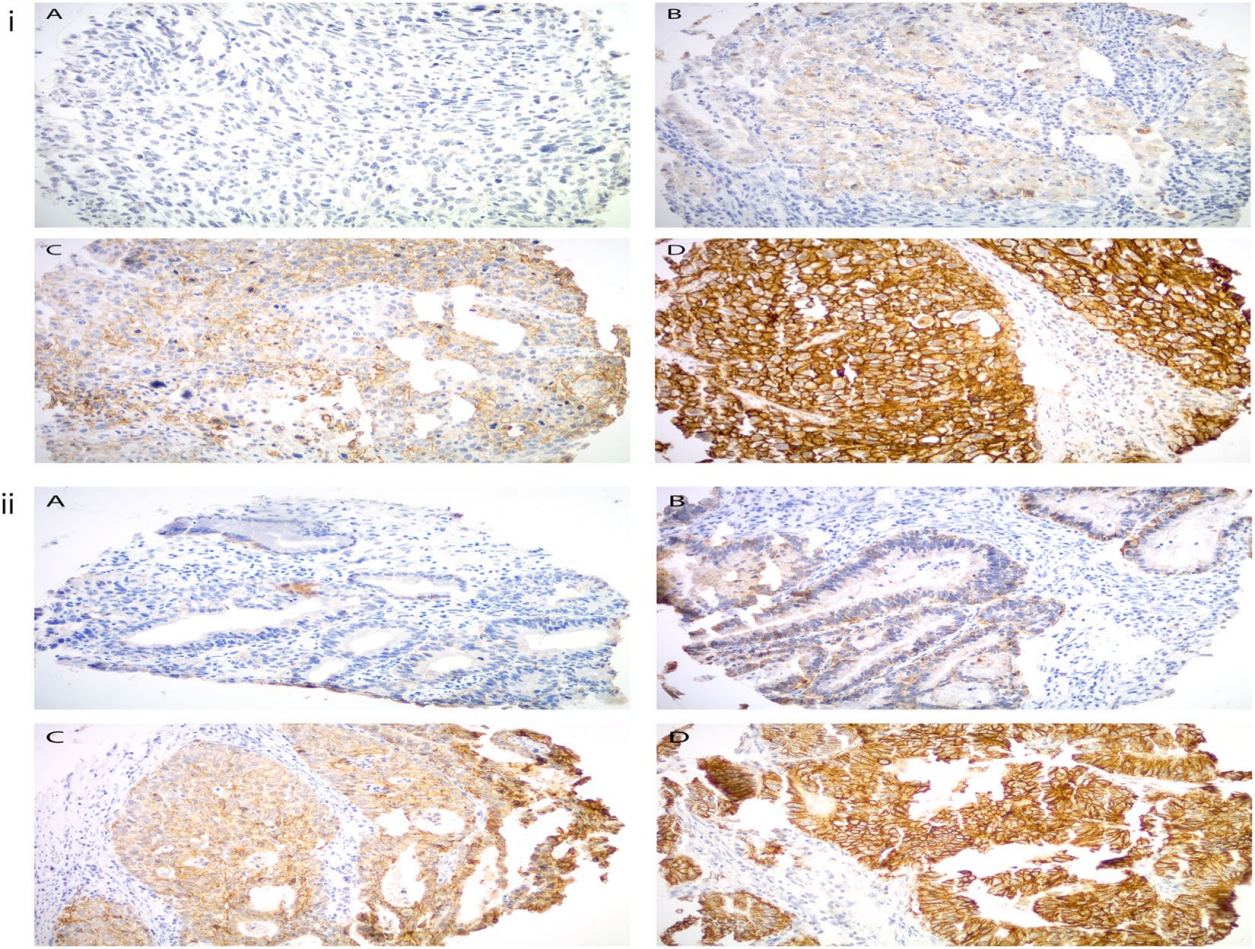
(i) and (ii), Trop2 expression by immunohistochemistry in cervical squamous cell carcinoma (upper panel) and cervical adenocarcinoma (lower panel), respectively. Representative images from the tissue microarray show (**A**) no Trop-2 immunostaining (score 0), (**B**) weak focal (score 1), (**C**) moderate focal (score 2), and (**D**) strong diffuse (score 3) Trop2 expression. All images at 200 × original magnification.

A TMA was used to semi-quantitatively analyze Trop-2 expression by IHC. Out of 147 primary cervical tumors, 113 were SCCs, and 34 were adenocarcinomas (n = 27) and adenosquamous cancers (n = 7). Moderate to strong diffuse staining was seen in 95% (108/113) of SCCs, and 81% (29/34) of adeno/adenosquamous carcinomas. In terms of strong diffuse staining (IHC score of 3), these rates were 71% (80/113) and 56% (19/34), respectively (supplementary table S2). Representative IHC images of the IHC score range are presented in Fig. 1.

### Trop-2 expression in primary cervical cancer cell lines by flow cytometry

**Figure 2:**
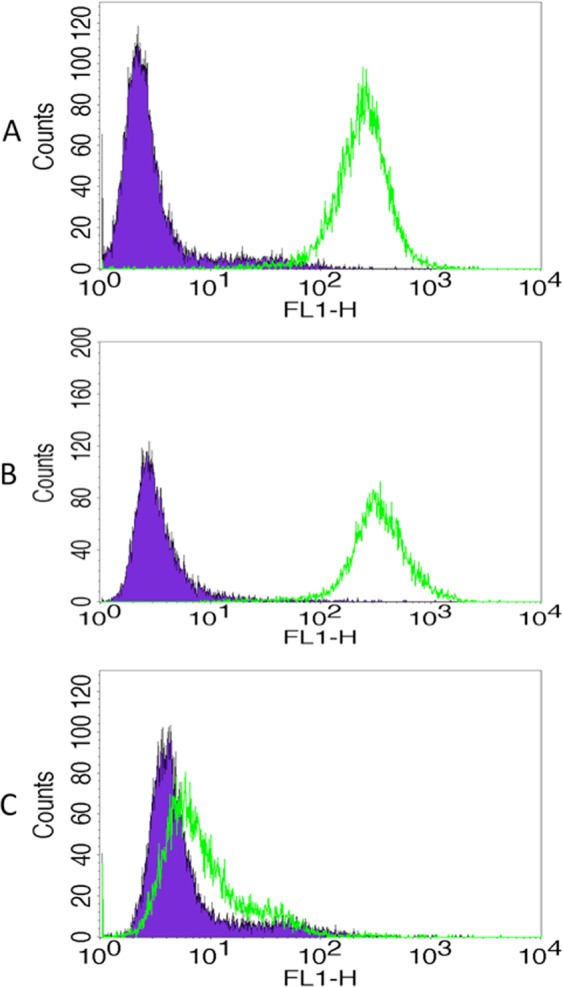
Representative flow cytometry histograms of primary cervical cancer cell lines. (**A**) CVX8, 2 + Trop-2 expression, (**B**) ADX3, 2 + Trop-2 expression, (**C**) ADX2, 0 Trop-2 expression.

Nine primary cervical cancer cell lines were established as described in Methods. Tumor characteristics including stage, grade, and histology are shown in supplemental data (Supplementary Table S1). Cell lines with a mean fluorescence index (MFI) greater than 50 were determined to have 2 + Trop −2 expression, those with an MFI of 20 to 50 were 1+, and those with 20 or less were 0. Eight-nine percent (8/9) of the cervical cancer cell lines were determined to have strong (2+) Trop-2 expression by flow cytometry. A representative flow cytometry histogram of three representative primary cervical cancer cell lines showing 2 + (CVX8, ADX3) and 0 (ADX2) Trop-2 expression, is shown in Fig. 2 (Supplementary Table S1 and Fig. 2).

### *In Vitro* Viability assays with sacituzumab govitecan, non-targeting control ADC and hRS7 IgG in primary cervical cancer cell lines

**Figure 3:**
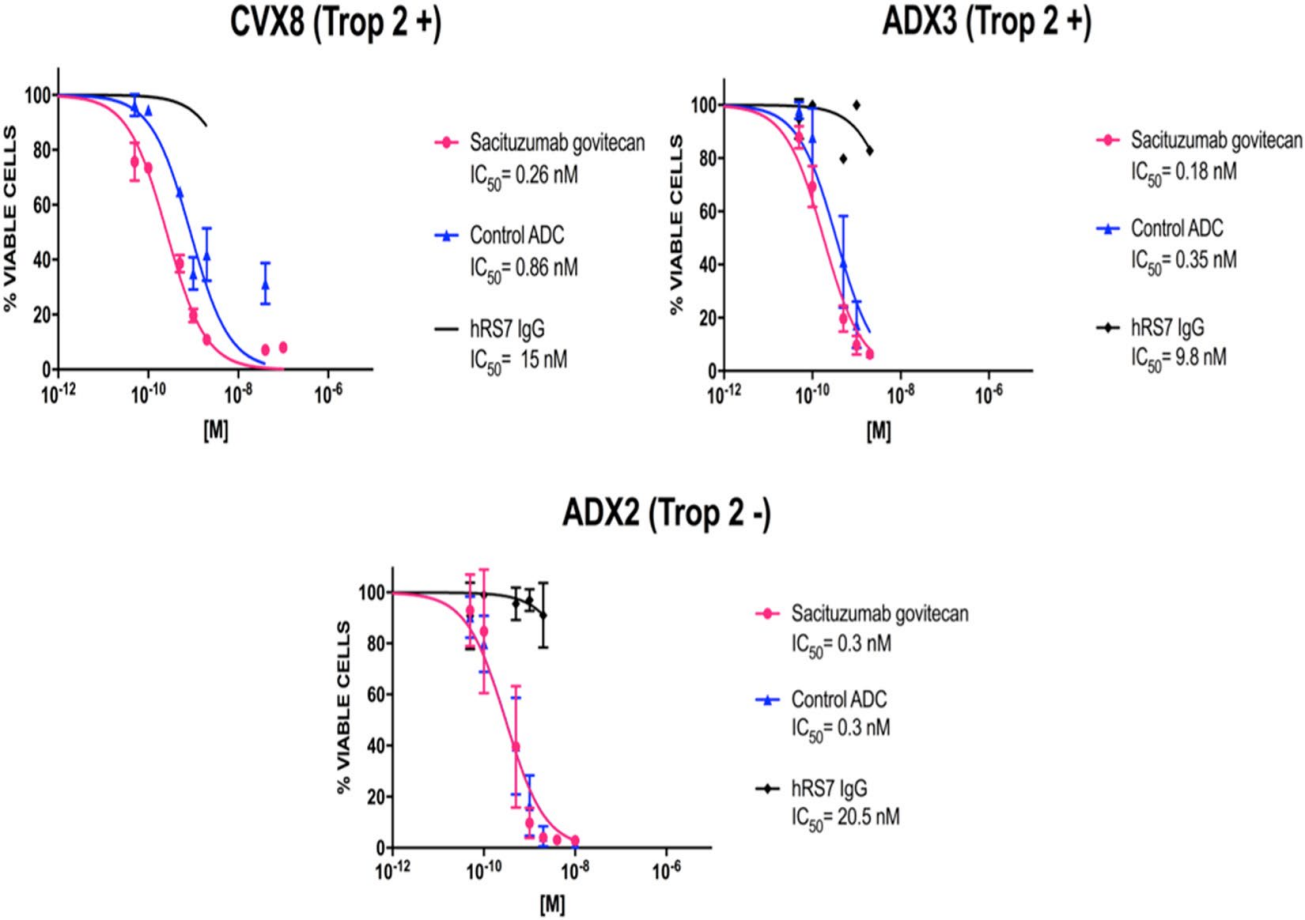
Determination by IC_50_ of sacituzumab govitecan cytotoxicity compared to controls, ADC isotype and hRS7 IgG in cervical cancer. (**A**) Cervical cancer cell line with high Trop-2 expression (2 + ) (CVX8) demonstrated significantly lower IC_50_ when compared to ADC isotype control (3.3 fold decrease, p < 0.05). (**B**) Cervical cancer cell line with high Trop-2 expression (2+) (ADX3) demonstrated significantly lower IC_50_ when compared to ADC isotype control (1.9 fold decrease, p < 0.05). (**C**) Cervical cancer cell line with low/negligible Trop-2 expression (ADX2) showed no difference in the IC_50_s of sacituzumab govitecan and ADC isotype control. hRS7 IgG antibody was inactive against these cell lines.

Three primary cervical cancer cell lines (ie, CVX8 and ADX3, Trop-2 positive and ADX2, Trop-2 negative) (Supplementary Table 1) were used for *in vitro* viability assays in various concentrations of sacituzumab govitecan (hRS7-CL2A-SN-38), non-targeting control ADC (h679-CL2A-SN-38), and naked antibody hRS7-IgG for a total of 3 days. The IC_50_ of sacituzumab govitecan, control ADC, and hRS7 IgG for each cervical cancer cell line was determined as described in Methods. Sacituzumab govitecan demonstrated significantly more potent cytotoxicity (ie, 3.3-fold and 1.9-fold increase in cell cytotoxicity against CVX8 and ADX3, respectively) when compared to the ADC isotype control in Trop-2 positive cell lines (p < 0.05) (Fig. 3). No difference in cytotoxicity was noted for the Trop-2 negative cell line (ADX2) (p = 0.9) (Fig. 3). The IC_50_ of hRS7 IgG (naked MAb) was found to be 55 to 68 fold higher than the IC_50_ of sacituzumab govitecan for all cell lines showing that naked MAb has no activity in the absence of effector NK cells in the *in vitro* studies.

### Sacituzumab govitecan and hRS7 IgG induce ADCC against Trop2-positive primary cervical cancer

**Figure 4:**
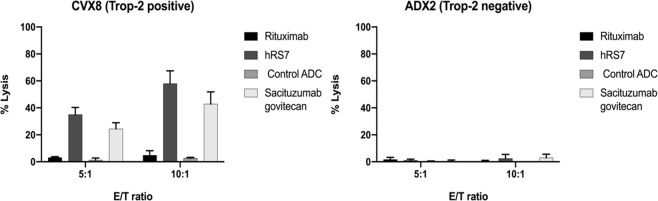
ADCC results (mean ± SEM) of sacituzumab govitecan, ADC isotype control, hRS7 IgG and Rituximab (anti-CD20) in two representative cervical cancer cell lines (ie, CVX8 2 + Trop-2 positive cell line vs ADX2 Trop-2 negative cell line). Significant ADCC was detectable only against the Trop-2 positive tumors.

Two representative primary cervical cancer cell lines (CVX8, 2 + Trop-2 positive and ADX2, Trop-2 negative) were tested for their sensitivity to PBL-mediated cytotoxicity as described in Methods. Both cell lines were consistently found to be resistant to PBL-mediated cytotoxicity when combined with PBLs and isotype control antibody (Rituximab) (2 μg/mL) at E:T ratios of 5:1 and 10:1 (mean ± SEM cytotoxicity of 2.36 ± 0.8% and 2.60 ± 2.2%) (Fig. 4). Sensitivity experiments were then performed in the presence of sacituzumab govitecan (hRS7-CL2A-SN-38), non-targeting control ADC (h679-CL2A-SN-38), and naked antibody hRS7-IgG at 2 μg/mL (Fig. 4). Trop-2 positive cell line (CVX8) induced ADCC at high levels in the presence of sacituzumab govitecan (hRS7-CL2A-SN-38) and hRS7-IgG (mean cytotoxicity ± SEM = 42.7% ± 5.2% for sacituzumab govitecan vs 57.9% ± 5.5% for hRS7-IgG, p = 0.12) but not h679-CL2A-SN-38 (non-targeting control ADC, mean cytotoxicity ± SEM = 2.6% ± 0.4%). In contrast, neither hRS7-IgG nor sacituzumab govitecan induced significant ADCC against Trop-2 negative cell line, ADX2 (Fig. 4).

### Bystander Effect *in vitro*

**Figure 5:**
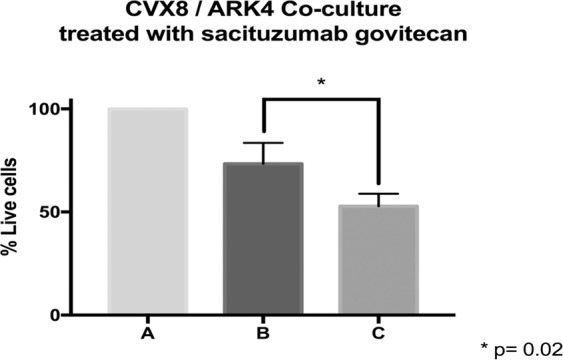
Bystander effect assay. (**A**) Low/negative Trop-2 expressing cells, ARK4 (GFP-ARK4 cells) co-cultured without any treatment (control), (**B**) Low Trop-2 expressing cells (ARK4 with GFP) co-cultured in 1:1 fashion with high Trop-2 expressing cells (CVX8), and treated with control ADC, (**C**) Low Trop-2 expressing cells (ARK4 with GFP) co-cultured in 1:1 fashion with high Trop-2 expressing cells (CVX8), and treated with sacituzumab govitecan. Significant increase in ARK4 cell cytotoxicity was detected at the time of the co-incubation with CVX8 cells (p = 0.02).

To evaluate the ability of sacituzumab govitecan to induce a bystander killing effect in a tumor environment where Trop-2 is expressed heterogeneously we tested the ADC activity by admixing CVX8 (ie, high Trop-2 expression) *in vitro* with low/negligible Trop-2 expressing cells (i.e., GFP-ARK4 cells) for 72 hours. As shown in Fig. 5, a significant increase in cytotoxicity of ARK4 cells was seen when ARK4 and CVX8 were cultured together and treated with sacituzumab govitecan when compared to ADC-control-treated ARK4 monocultures (p = 0.02).

### *In vivo* antitumor activity

**Figure 6:**
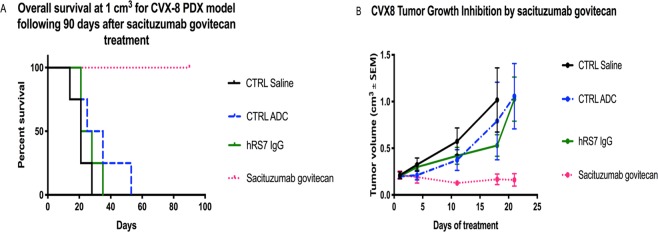
*In vivo* efficacy of sacituzumab govitecan: Antitumor activity of sacituzumab govitecan was compared to controls including, ADC isotype, hRS7 IgG and saline, in cervical cancer xenograft models (ie, CVX8, 2 + Trop-2 positive). Mice were treated intravenously with twice-weekly doses for three weeks as described in Methods. (**A**) Overall survival. (**B**) Tumor growth inhibition. A significant difference in tumor growth inhibition was detected beginning on day 18 (p < 0.001) in sacituzumab govitecan-treated group when compared to the other control groups.

For *in vivo* experiments, we compared the antitumor activity of sacituzumab govitecan (hRS7-CL2A-SN-38), control ADC (h679-CL2A-SN-38), naked hRS7 IgG and normal saline (four mice in each group) against Trop-2 positive cervical cancer xenografts (CVX8). All treatments were given twice per week for three weeks by intravenous injection of 500 μg of sacituzumab govitecan, control ADC, saline and hRS7 IgG. Sacituzumab govitecan significantly inhibited tumor growth when compared to control ADC, saline and hRS7 IgG beginning at day 18 of the treatment (p < 0.0001, and p = 0.001 for sacituzumab govitecan vs naked antibody, and sacituzumab govitecan vs control ADC, respectively). This protective effect was evident for the entire treatment period as well as during the 90- day follow-up period, while mice treated with saline, control ADC (h679-CL2A-SN-38), and hRS7 IgG all demonstrated rapid tumor growth (Fig. 6). Overall survival at 90 days was significantly improved in the sacituzumab govitecan group (p = 0.014) (Fig. 6). Animals tolerated sacituzumab govitecan treatment well with no significant toxicity.

## Discussion

Trop-2 was first described on human trophoblast cells as a calcium signal transducer, comprised of a large extracellular domain, a single transmembrane domain, and a short cytoplasmic tail. However, its expression, role, and function became of great interest only few years later, after the discovery of Trop-2 overexpression on the surface of many different types of epithelial tumors^8^.

Liu *et al*. recently investigated the effect of gain-or-loss of Trop-2 expression through ectopic overexpression or RNA interference mediated knockdown in primary cervical cancer cell lines^18^. Trop-2 was able to promote cell proliferation by inhibiting apoptosis and accelerating cell cycle progression. Inhibition of apoptosis was mediated through increased expression of bcl-2, together with decreased expression of bax while down-regulation of Trop-2 induced arrest in G1 phase of the cell cycle. These results are in agreement with the oncogenic effect of Trop-2 identified in multiple other epithelial human tumors^8^.

In the present study, we examined Trop-2 expression in large number of cervical cancers. We found the majority of squamous, adenocarcinoma and adenosquamous cancers to overexpress Trop-2. Moreover, 8/9 (89%) of the cell lines tested in our study were Trop-2 positive by flow cytometry, further supporting Trop-2 as a potential target in cervical cancer patients. Next, we investigated to our knowledge for the first time, the *in vitro* and *in vivo* activity of sacituzumab govitecan, a novel ADC that couples a humanized monoclonal Trop-2 antibody and SN-38 (active metabolite of irinotecan) with a cleavable linker that is subject to time-dependent hydrolysis^7^, against primary cervical cancer cell lines and xenograft models. We report significant differential sensitivity to sacituzumab govitecan *in vitro* when compared to ADC isotype and unconjugated antibody controls in all Trop-2 positive cervical cancer cell lines tested. In contrast, no significant differences in cytotoxicity were detected after the exposure to sacituzumab govitecan and ADC isotype control in Trop-2 negative primary cervical cancer cell lines. These data clearly demonstrate the specificity of sacituzumab govitecan killing against tumor cells overexpressing the TROP-2 target. In our *in vitro* experiments in Trop-2 positive cell lines, the differential killing between sacituzumab govitecan vs the matched non-targeting control ADC ranged from 1.9 to 3.3 folds. We believe the relative low differential *in vitro* activity (making the demonstration of specificity difficult) is related to the rapid release of SN-38 (ie, the toxic payload of sacituzumab govitecan and the control ADC) from the conjugate when the ADCs are incubated in culture medium containing serum at 37 °C (ie, half-life 24 hrs)^8^.

Importantly, both sacituzumab govitecan and hRS7-IgG demonstrated significant ADCC against Trop-2 positive cell lines *in vitro* while negligible ADCC was detected after exposure of the same primary tumor cell lines to the non-targeting control ADC. These data confirm the results of a previous study performed by our group, in which Trop-2 expressing cervical cancer cell lines were found highly susceptible to ADCC when exposed to the naked humanized anti-Trop-2 monoclonal antibody hRS7^17^. Taken together, our study suggests that the *in vitro* cytotoxic effect of sacituzumab govitecan is greater in Trop-2 positive cells.

The current standard treatment of recurrent, advanced or metastatic cervical cancer has emerged from a randomized, open-label, phase III trial, conducted by Gynecologic Oncology Group (protocol 240), in which two independent treatment modalities (non-platinum chemotherapy doublet – topotecan/paclitaxel - and incorporation of anti-angiogenesis therapy) were investigated against standard of care regimen consisting of cisplatin (50 mg/m^2^) and paclitaxel (135 mg/m^2^)^19^. Final results demonstrated that addition of anti-angiogenesis agent, bevacizumab, to chemotherapy regimens resulted in improved overall survival (OS) (median, 17.0 versus 13.3 months, hazard ratio [HR] 0.77, 98% CI 0.54–0.95), progression free survival (PFS) (median, 8.2 versus 5.9 months; HR 0.67, 95% CI 0.54–0.82), and overall response rate (ORR) (49% versus 36%). Unfortunately, once patients progress after this initial combined therapy for recurrent or metastatic disease, effective options of treatment are very limited.

Sacituzumab govitecan ADC may therefore represent a novel, potentially effective treatment option for cervical cancer patients with recurrent/metastatic disease resistant to standard treatment modalities. While no published data currently exists demonstrating the clinical activity of sacituzumab govitecan in cervical cancer patients, few papers have recently reported the *in vivo* activity of sacituzumab govitecan in patients harboring a variety of epithelial tumor types overexpressing Trop-2^8^. In the dose escalation phase of the phase I/II basket trial reported by Starodub *et al*., patients received 2 consecutive doses of sacituzumab govitecan on days 1 and 8 of a 3-week treatment cycle and could continue treatment for up to 8 cycles in the absence of toxicity or progression and based on improved tolerability to additional cycles and observed clinical activity, the 8 and 10 mg/kg doses were selected for dose expansion^7^. Results from the phase II dose expansion portion identified the 10 mg/kg dose for further evaluation^20^. Findings from the individual cohorts from the phase I/II basket study including breast, urothelial, non-small cell and small cell lung cancer patients showed overall response rates ranging from 14% to 33%^9–13^. In the cohort of 108 patients with triple negative breast cancer, who had received at least 2 prior lines of chemotherapy (median 3, range 2–10), the response rate (3 complete and 33 partial responses) was 33.3% (95% confidence interval [CI], 24.6 to 43.1), and the median duration of response was 7.7 months (95% CI, 4.9 to 10.8)^9^. Adverse events leading to interruption of treatment occurred in 44% of the patients. The rate of serious adverse events was 32%, the most common (>2% incidence) being neutropenia (7%), which was followed by vomiting (6%), nausea (4%), diarrhea (3%), and dyspnea (3%)^9^.

One major challenge in the treatment of cancers using targeted therapeutics such as monoclonal antibodies stems from the recent evidence that many gynecologic tumors demonstrate high heterogeneity in the expression of target antigens. For example, in uterine serous carcinoma (USC), a biologically aggressive endometrial tumor, whole exome sequencing (WES) and confirmatory IHC studies revealed that up to 35% of cases harbor human epidermal growth factor receptor 2 (HER2) overexpression, and over 50% of patients overexpressing HER2 at 3 + levels demonstrated high heterogeneity (at least two-degree difference in staining intensity involving tumor cells)^16,21^. This knowledge provided a potential molecular explanation to the failure of previous NRG/NCI cooperative clinical trials targeting endometrial cancer patients with single-agent trastuzumab (a humanized monoclonal antibody targeting HER2/neu)^22^. More importantly, a better understanding of endometrial cancer heterogeneity was paramount for the design and implementation of the first successful study with trastuzumab in USC patients with advanced/recurrent disease overexpressing HER2/neu^23^. Challenges with antigen heterogeneity in tumors may at least partially be overcome by using ADCs with cleavable linkers, which are active not only against tumor antigen positive target cells but also against tumor antigen negative surrounding cells through a bystander killing effect^5,6^. Bystander killing may occur when cytotoxic agents linked to an antibody are released either from the target cells following internalization and subsequent lysosomal degradation of the ADC or when drug is released within the extracellular space after reaching the tumor microenvironment by a cleavable pH sensitive linker^5,6^. Consistent with this view, we experimentally demonstrated that sacituzumab govitecan induced bystander killing of Trop-2 negative tumor cells when admixed with Trop-2 overexpressing tumor cells. These results strongly suggest that sacituzumab govitecan may be active in the treatment of recurrent cervical cancer patients harboring tumors with heterogeneous Trop-2 expression.

## Conclusions

In summary, our results demonstrate that (1) Trop-2 is overexpressed in the majority of both squamous cell and adenocarcinomas of the cervix, (2) primary cervical cancer cell lines overexpressing Trop-2 are highly susceptible to killing *in vitro* by sacituzumab govitecan, (3) sacituzumab govitecan in the presence of effector cells (NK cells) may induce significant ADCC against Trop-2 positive cervical cancer cells (4) sacituzumab govitecan demonstrated a significant bystander killing effect, which could aid in treating tumors with heterogeneous antigen expression and (5) most importantly, cervical cancer xenografts overexpressing Trop-2 are highly sensitive *in vivo* to sacituzumab govitecan. These preclinical results combined with the recent phase II data demonstrating significant clinical responses in multiple solid tumors that are resistant to chemotherapy, strongly support the design of clinical trials in Trop-2 positive recurrent or advanced cervical cancer patients.

## Supplementary information

Supplementary Information

## Data Availability

The datasets used and/or analyzed during the current study are available from the corresponding author on reasonable request.
