# Supplementary material for: Cervical carcinomas that overexpress human trophoblast cell-surface marker (Trop-2) are highly sensitive to the antibody-drug conjugate sacituzumab govitecan
Source: Sci Rep. 2020 Jan 22;10:973. doi: 10.1038/s41598-020-58009-3 (PMC6976591; doi:10.1038/s41598-020-58009-3)
Supplement: Supplementary file 1 — Supplementary Information [file 41598_2020_58009_MOESM1_ESM.docx]

**Cervical carcinomas that overexpress human trophoblast cell-surface marker (Trop-2) are highly sensitive to the antibody-drug conjugate sacituzumab govitecan**

Burak Zeybek^1^, Aranzazu Manzano^1^, Anna Bianchi^1^, Elena Bonazzoli^1^, Stefania Bellone^1^, Natalia Buza^2^, Pei Hui^2^, Salvatore Lopez^3^, Emanuele Perrone^1^, Paola Manara^1^, Luca Zammataro^1^, Gary Altwerger^1^, Chanhee Han^1^, Joan Tymon-Rosario^1^, Gulden Menderes^1^, Elena Ratner^1^, Dan-Arin Silasi^1^, Gloria S. Huang^1^, Masoud Azodi^1^, Peter E. Schwartz^1^, Alessandro Santin^1^

^1^Department of Obstetrics, Gynecology, and Reproductive Sciences and ^2^Department of Pathology, Yale University School of Medicine, CT 06520, ^3^Department of Experimental and Clinical Medicine, Magna Graecia University, Catanzaro, 88100, Italy

***Address for Correspondence:*** Alessandro D Santin, MD

Department of Obstetrics, Gynecology, and Reproductive Sciences

Co-Chief Gynecologic Oncology

Yale School of Medicine

LSOG Bld. Room 305, 333 Cedar Street, PO Box 208063, New Haven, CT, 06520-8063.

Phone: 203-737-4450

Fax: 203-737-4339

Email: alessandro.santin@yale.edu

**Supplementary Table S1:** Cervical cancer cell lines with demographics, stage, histologic grade, primary site of tumor, Mean fluorescence intensity (MFI) and score for Trop-2. International Federation of Gynecology and Obstetrics (FIGO) staging and grading.

| Cell line | Age | Ethnicity | FIGO stage | Histology | Flow Cytometry (MFI) | Trop-2  Score |
| --- | --- | --- | --- | --- | --- | --- |
| ADX1 | 40 | White | IB | Squamous cell | 205.41 | +2 |
| ADX3 | 25 | White | IB | Adenocarcinoma | 202.90 | +2 |
| CVX1 | 40 | White | IB | Squamous cell | 43.51 | +1 |
| CVX3 | 35 | African-American | IB | Squamous cell | 97.05 | +2 |
| CVX4 | 40 | White | IIA | Squamous cell | 378.17 | +2 |
| CVX5 | 42 | White | IB | Squamous cell | 220.37 | +2 |
| CVX8 | 29 | White | IB | Squamous cell | 210.81 | +2 |
| ADX2 | 33 | African-American | IB | Adenocarcinoma | 7.97 | 0 |

**Supplementary Table S2:** Immunohistochemistry scores based on histologic type

|  | Squamous cell carcinoma (n=113)  N (%) | Adenocarcinoma  (n=27)  N (%) | Adenosquamous  (n=7)  N (%) |
| --- | --- | --- | --- |
| IHC Score 0 | 2 (1.8%) | 2 (7.4%) | 0 |
| IHC Score 1 | 3/113 (2.6%) | 3 (11.1%) | 0 |
| IHC Score 2 | 28/113 (24.8%) | 10 (37%) | 0 |
| IHC Score 3 | 80/113 (70.8%) | 12 (44.4%) | 7 (100%) |
